# Local administration of large surface area microparticle docetaxel to solid carcinomas induces direct cytotoxicity and immune-mediated tumoricidal effects: preclinical and clinical studies

**DOI:** 10.1007/s13346-022-01226-2

**Published:** 2022-09-04

**Authors:** Holly Maulhardt, Shelagh Verco, Michael Baltezor, Alyson Marin, Gere diZerega

**Affiliations:** 1 US Biotest, Inc, 231 Bonetti Drive, Suite 240, San Luis Obispo, CA 93401 USA; 2 CritiTech, Inc, 1849 E 1450 Road, Lawrence, KS 66044 USA; 3 NanOlogy, LLC, 3909 Hulen Street, Fort Worth, TX 76107 USA

**Keywords:** LSAM-DTX, Intratumoral injection, Docetaxel, Urinary bladder cancer, Breast cancer, Immunomodulation

## Abstract

This report describes local administration of large surface area microparticle docetaxel (LSAM-DTX: ~ 3.5- to 7.5-µm-sized particles with high relative surface area) in preclinical oncology models and in a clinical trial in urothelial carcinoma. Reductions in tumor volumes were found following intratumoral (IT) injection of LSAM-DTX into human urologic carcinoma cell lines and syngeneic murine renal and breast cancer cell lines. Compared to IT injections of docetaxel solution typically administered intravenously, IT LSAM-DTX results in 40-fold more docetaxel retained within the tumor. The long residence time of LSAM-DTX within the tumor acts as a drug depot, allowing for continuous release of docetaxel, exposing tumor cells to high, therapeutic levels of chemotherapeutic for several weeks. Local LSAM-DTX results in tumoricidal effects at the site of deposition as well as in distant tumors, and IT LSAM-DTX in combination with immune checkpoint inhibitor therapy reduces or eliminates metastatic spread. Tumoricidal effects of local LSAM-DTX are accompanied by immunomodulation including increases in innate and adaptive immune cells in the tumor microenvironment and peripheral blood. Encouraging clinical results indicate that local administration of LSAM-DTX may provide therapeutic benefits for non-muscle invasive bladder cancer and muscle invasive bladder cancer patients; treatments were well-tolerated with few local and systemic adverse events and negligible systemic docetaxel exposure. Results of preclinical and clinical investigations summarized here indicate that local administration of LSAM-DTX may augment tumor response to systemically administered chemotherapy, targeted therapy, or immunotherapy without contributing to systemic toxicity.

**Graphical abstract:**

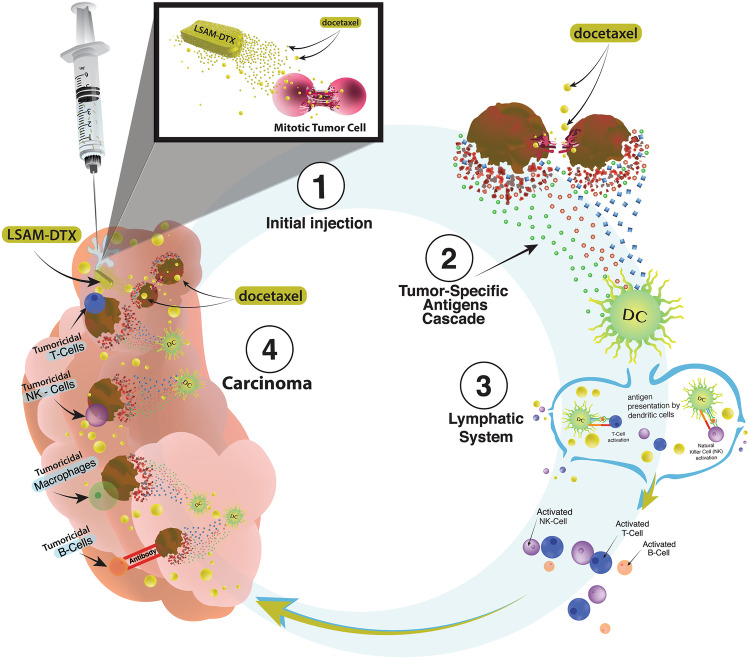

## Introduction

Local treatment of solid tumors with chemotherapy has the potential to overcome the limitations of conventional intravenous (IV) administration. These limitations include rapid systemic distribution which results in reduced intratumoral (IT) concentrations of chemotherapy and tumor dwell time, as well as increased systemic toxicities [1]. Treatment of solid carcinomas would likely be improved by continuous exposure of tumor cells to therapeutic taxane drug concentrations over multiple cell-division cycles [2]. IT injection of particles of anti-neoplastic drugs, such as taxanes, a class of antineoplastic drugs commonly prescribed since early 1990s [3], would reduce systemic toxicity, thus providing tumoricidal benefits without dose-limiting toxicity. Investigations of large surface area microparticle paclitaxel (LSAM-PTX) in preclinical and clinical studies treating solid carcinomas have been described previously [4]. Clinical trials of LSAM-PTX for treatment of lung cancer (NCT04314895) and locally advanced pancreatic cancer (NCT03077685) are ongoing [5]. Clinical trials of LSAM-PTX have been completed in peritoneal cancers (NCT00666991 [6]; NCT03029585 [7]), prostate cancer (NCT03077659) [4], and mucinous cystic pancreatic neoplasms (NCT03188991) (Table 1). In addition, local treatment of the primary tumor may enhance response to immunotherapy at the tumor site and could stimulate systemic anti-cancer immune responses [8].

**Table 1:** Clinical trials of large surface area microparticle (LSAM) taxanes

**Study ID**	**NCT number**	***n***	**Route of administration**	**Study title**	**Study status**^a^
**LSAM Docetaxel (LSAM-DTX)**					
NANODOCE-2017–02	NCT03636256	36	Direct injection to the bladder wall and intravesical instillation	Phase 1/2 Trial Evaluating the Safety and Tolerability of NanoDoce^®^ Injection and Intravesical Instillation in Subjects With Urothelial Carcinoma [12]	Completed
**LSAM Paclitaxel (LSAM-PTX)**					
HSC#1114	NCT00666991	21	Intraperitoneal	Pharmacokinetic, Safety and Efficacy Study of Nanoparticle Paclitaxel in Patients with Peritoneal Cancers [6]	Completed
NANOPAC-2016–01	NCT03029585	10	Intraperitoneal	Phase II Study of Four Dose Levels of Intraperitoneal NanoPac Plus IV Carboplatin and Paclitaxel in Patients With Epithelial Ovarian Cancer Undergoing Cytoreductive Surgery [7]	Completed
NANOPAC-2016–02	NCT03077659	16	Intraprostatic injection	Phase IIa Dose Escalation Trial of NanoPac Focal Therapy for Prostate Cancer in Subjects Undergoing Radical Prostatectomy [4]	Completed
NANOPAC-2017–01	NCT03188991	19	Intracystic injection	A Trial Evaluating Escalating Doses and the Safety of Intracystic Injection of NanoPac in Subjects with Mucinous Cystic Pancreatic Neoplasms	Completed
NANOPAC-2016–05	NCT03077685	54	Intratumoral injection	Phase IIa Trial Evaluating the Safety of Intratumoral Injection of NanoPac in Subjects with Locally Advanced Pancreatic Adenocarcinoma	Active, not recruiting
NANOPAC-2019–01	NCT04221828	1	Intratumoral injection	Phase 2 Trial of NanoPac Focal Therapy for Prostate Cancer in Subjects Undergoing Radical Prostatectomy	Terminated
NANOPAC-2020–01	NCT04314895	18	Intratumoral injection	Phase 2 Trial Evaluating the Safety and Tolerability of Intratumoral Injections of NanoPac^®^ with Standard of Care Therapy in Subjects with Lung Cancer	Active, not recruiting

^a^Study status current as of 4 August 2022

This paper summarizes recent preclinical results [9–11] evaluating IT administration of large surface area microparticle docetaxel (LSAM-DTX) in a variety of tumor types alone and in combination with immunotherapy and the initial clinical trial utilizing local administration of LSAM-DTX to treat high-risk non-muscle invasive bladder cancer (NMIBC) and muscle-invasive bladder cancer (MIBC) (NCT03636256) [12].

Discussion is provided regarding differences in tumor response and immunomodulation following IV chemotherapy vs. IT injection of LSAM-taxanes. In addition, follow-on clinical applications of the technology are considered.

## Preparation of submicron particles

**Fig. 1:**
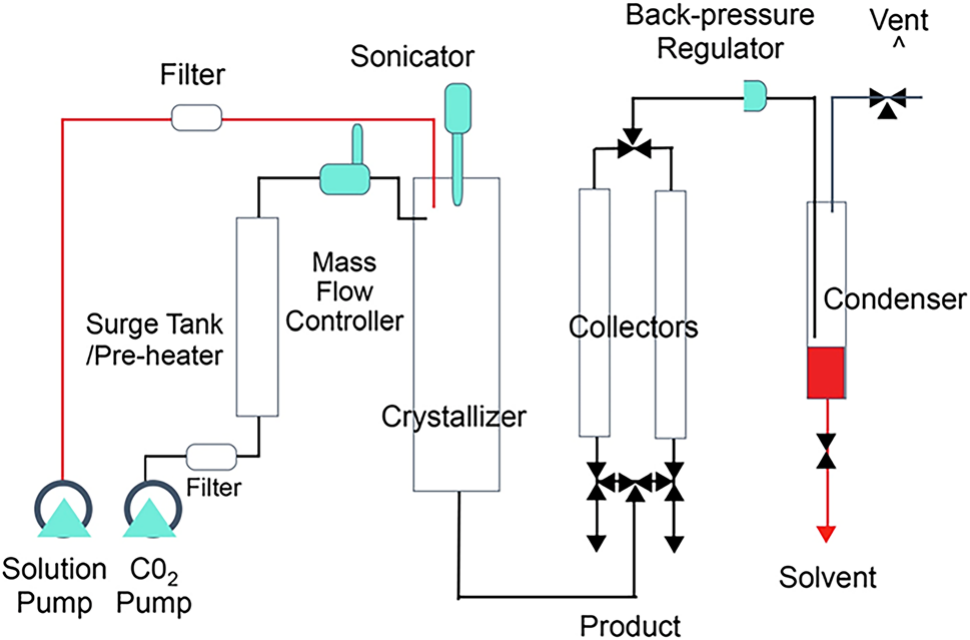
Schematic of manufacturing process for large surface area submicron particle docetaxel (LSAM-DTX). Supercritical carbon dioxide (ScCO_2_) above the critical point (> 72.8 bar, > 31 °C) is utilized in the manufacturing process. ScCO_2_ is miscible with organic solvents but a poor solvent for docetaxel which is relatively hydrophobic. Mixing docetaxel in organic solvent with scCO_2_ causes LSAM-DTX to precipitate into small particles. These small particles are captured in a filter and dried. LSAM-DTX particle size distribution is determined using a validated analytical method in which particles are suspended in 0.01% lecithin in hexane (non-solvent) and analyzed by laser diffraction on a Mastersizer 3000 particle size analyzer (Malvern Panalytical Ltd., Malvern, UK)

**Fig. 2:**
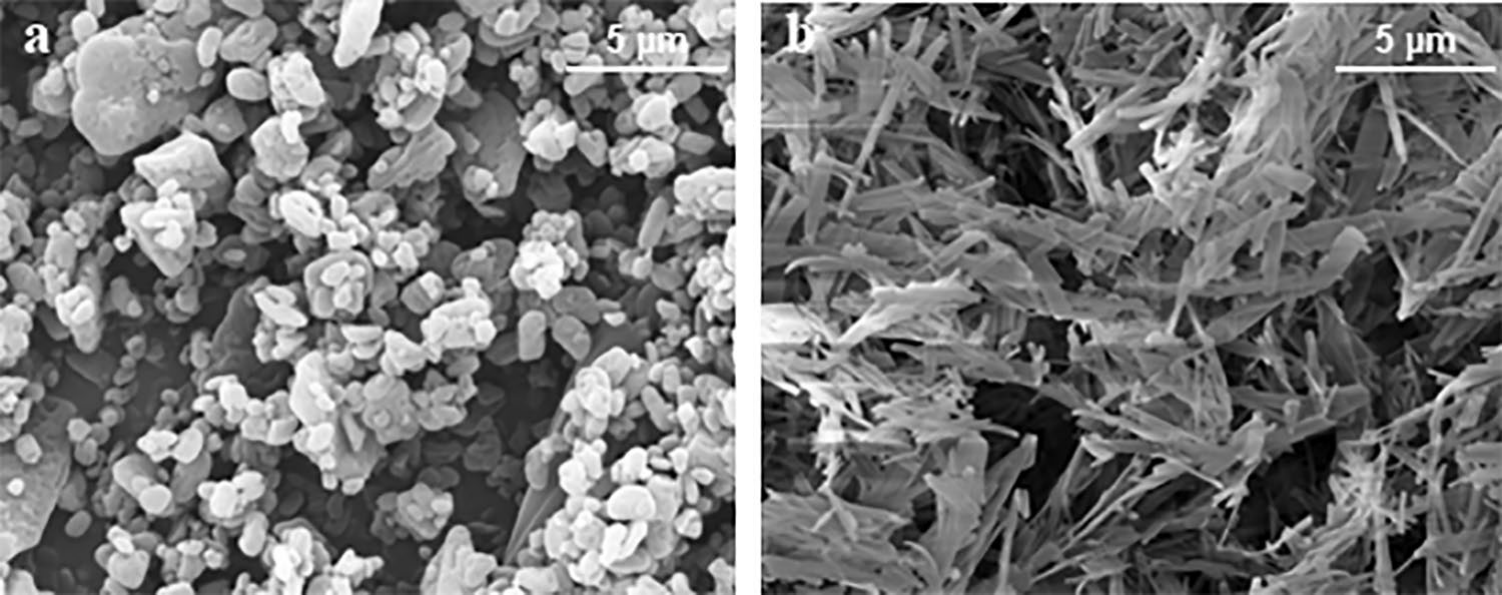
**a** Electron micrograph of docetaxel drug substance prior to processing with specific surface area (SSA) = 6.98 m^2^/gram and median particle volume (Dv50) = 2.85 µm. **b** Electron micrograph of large surface area microparticle docetaxel (LSAM- DTX) with SSA = 25.83 m^2^/g and Dv50 = 3.89 µm

Particles with a large surface area relative to weight and size comprised only of taxanes (docetaxel: LSAM-DTX, NanoDoce^®^ or paclitaxel: LSAM-PTX, NanoPac^®^, Nanotax^®^; CritiTech, Inc., Lawrence, KS) were developed to increase IT drug residence time to provide continuous delivery of taxane molecules through multiple mitotic cycles of tumor cells. LSAM-DTX are produced by proprietary technology that sonicates docetaxel drug substance dissolved in a suitable organic solvent into uniform droplets (Fig. 1) [13]. The solvent is instantaneously stripped from the solution using supercritical fluid carbon dioxide and sonic energy resulting in the precipitation of pure particles of docetaxel that have a volume-based mean particle size between approximately 3.5 and 7.5 µm. As the LSAM name implies, the particles have a large specific surface area (SSA) which is a measure of the particle surface area-to-mass ratio and is directly proportional to the rate of drug release from the particles. Unlike traditional solid drug particles which increase surface area by particle size reduction, LSAM particles increase the surface area as part of the internal matrix structure. As an example, the LSAM-DTX particles shown in Fig. 2 had a mean particle size of 3.89 µm with a relative standard deviation of 2.7%. The SSA was 25.83 m^2^/g. In order to achieve this same high surface area by size reduction of the drug substance, the particle size would have to be < 0.8 µm. Besides the difficulty of achieving submicron particles by milling without the addition of surfactants to reduce agglomeration, smaller drug particles would have greater mobility and probability of removal from the tumor microenvironment (TME). LSAM-DTX particles are suspended at the time of use in a saline-based solution that can be delivered directly to the disease site using commonly employed techniques, including IT injection into solid carcinomas and intravesical instillation into the urinary bladder.

## Preclinical studies

### Tumor retention study

**Fig. 3:**
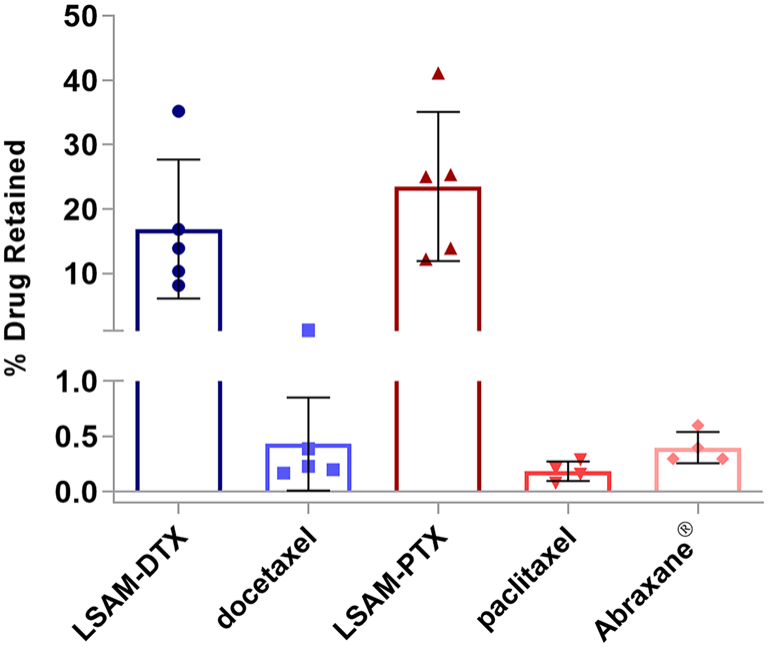
LSAM-taxanes increase intratumoral drug concentrations. Percent drug retained in MDA-MB-231 tumors. All formulations evaluated were prepared at 5-mg/mL concentrations and included suspensions of large surface area microparticle docetaxel (LSAM-DTX), large surface area microparticle paclitaxel (LSAM-PTX) or Abraxane^®^ (Nab-paclitaxel), or solutions of docetaxel or paclitaxel. Formulations were administered as 0.05-mL direct injections into MDA-MB-231 tumors implanted on the flanks of immunodeficient (NCr*-nu/nu*) female mice (*n* = 4 or 5 per group) and tumor tissues were collected 5 days later. The percent of drug retained is based on the concentration of drug detected (ng/g of tumor) and tumor weight divided by the nominal amount of drug delivered (250 ng/injection). Group mean docetaxel percent (blue bars) and paclitaxel percent (red bars) as well as individual animal tumor percents (symbols) are plotted; error bars =  ± 1 SD. Adapted with permission from [4]

The large size and irregular morphology of the LSAM-DTX particles decrease the mobility of particles or aggregates of particles after injection into the TME. The longer residence time and higher surface area increase the dissolution of drug from the LSAM-DTX particles resulting in continuous release of tumoricidal levels of chemotherapy for extended durations. A study was performed to determine the concentration of docetaxel or paclitaxel in a subcutaneous xenograft implant model of human breast adenocarcinoma (MDA-MB-231 cell line (ATCC^®^ HTB-26™) in nude (NCr *nu/nu*) female mice) following IT administration of 0.25 mg of either LSAM-DTX, LSAM-PTX, Nab-paclitaxel suspension, docetaxel solution, or paclitaxel solution (Table 2) [4]. Five days following IT administration of LSAM-DTX, the mean concentration of docetaxel in tumor tissues was approximately 140,000 ng/g of tissue compared to tumors from animals administered IT docetaxel solution which was approximately 4000 ng/g of tissue. These concentrations represent 16.9% (*n* = 5) of nominal docetaxel retained in the IT LSAM-DTX-treated tumor versus 0.4% (*n* = 5) of docetaxel retained following IT injection of a docetaxel solution (Fig. 3). Tumors administered IT LSAM-DTX retained 40-fold more docetaxel compared to tumors injected with docetaxel solution. Tumors administered IT LSAM-PTX retained similarly high levels of paclitaxel compared to Nab-paclitaxel suspensions (46-fold increase) or paclitaxel solution (128-fold increase).

**Table 2:** Preclinical studies of intratumoral (IT) large surface area microparticle docetaxel (LSAM-DTX)

**Tumor type**	**Cell line**	**Species/strain**^a^	***n, range ***	**IT LSAM-DTX dose (mg/kg)**^b^	**Cycles adminstered (cycle time)**	**Reference**
**Bladder**						
Human	UM-UC-3 (ATCC^®^ CRL-1749™)	Mouse/nude (Hsd:Athymic Nude-*Foxn1*^*nu*^)	9-10	100	1, 2, or 3 (weekly)	[9]
**Breast**						
Human	MDA-MB-231 (ATCC^®^ HTB-26 ™)	Mouse/nude (NCr *nu/nu*)	5	12.5	1	[4]
Mouse	4T1 (ATCC^®^ CRL-2539™)	Mouse/BALB/c (BALB/cAnNHsd)	7	50	4 (4 days)	[11]
**Prostate**						
Human	PC-3 (ATCC^®^ CRL-1435™)	Mouse/nude (NCr *nu/nu* (Crl:NU(NCr)-*Foxn1*^*nu*^)	10	37.5, 100	1 or 3 (weekly)	[9]
**Renal**						
Human	786-O (ATCC^®^ CRL-1932™)	Rat/Sprague–Dawley (Rag2/Il2rg null) SRG™	3	20	1, 2, or 3 (weekly)	[9]
Mouse	Renca (ATCC^®^ CRL-2947™)	Mouse/BALB/c (BALB/cAnNCrl)	10-15	27.5^c^, 55^c^	3 (weekly)	[10]

^a^All studies utilized female animals

^b^LSAM-DTX dose is approximated based on fixed volume IT injection not adjusted for individual animal body weight

^c^LSAM-DTX administered as multiple injections IT or IT + peritumoral (IT/PT)

### Improved efficacy in uro-oncologic xenografts

**Fig. 4:**
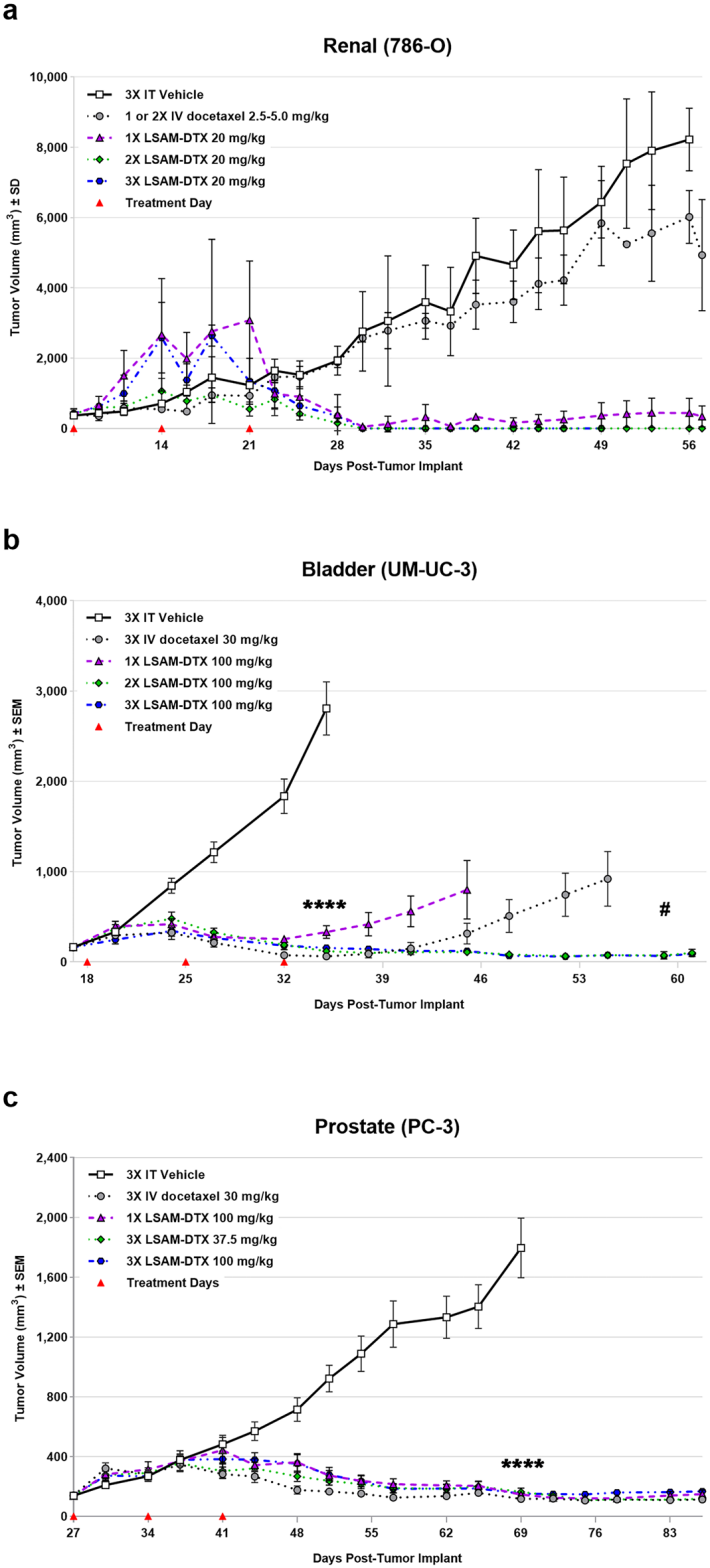
Improved efficacy in urologic carcinoma xenografts administered intratumoral (IT) large surface area microparticle docetaxel (LSAM-DTX) compared to intravenous (IV) docetaxel. **a** 786-O (ATCC^®^ CRL-1932™) xenografts in immunodeficient Sprague–Dawley (Rag2/Il2rg null (SRG™) female rats; *n* = 3/group; treatment was initiated 7 days after tumor implant when group mean TVs ranged from 336 to 427 mm^3^. IV docetaxel administered for two cycles of 5 mg/kg to 2 of 3 animals resulted in one immediate death due to cessation of respiration and one animal with temporary loss of respiration and prolonged recovery from anesthesia. Due to this toxicity, the third animal in the group was not administered a second cycle of IV docetaxel and the final cycle administered to both remaining animals was reduced to 2.5 mg/kg. Data plotted through point when ≥ 50% of animals in the group survived. Due to small number of animals per group, statistical analysis was not performed. **b** UM-UC-3 (ATCC^®^ CRL-1749™) xenografts in immunodeficient (Hsd:Athymic Nude-*Foxn1*^*nu*^) female mice; *n* = 9 or 10/group; treatment was initiated 18 days after tumor implant when group mean TVs ranged from 161 to 164 mm^3^. ****p < 0.0001 for all treatment groups vs. 3 × IT vehicle (day 34); ^#^*p* < 0.01 for 3 × IV docetaxel vs. 2 × and 3 × LSAM-DTX (Day 59). Data plotted through point when first animal in the group died. **c** PC-3 xenografts (ATCC^®^ CRL-1435™) in immunodeficient (NCr *nu/nu* (Crl:NU(NCr)-*Foxn1*^*nu*^)) female mice; *n* = 10/group; treatment was initiated 26 days after tumor implant when group mean TVs ranged from 136 to 141 mm.^3^. ****p < 0.0001 for all treatment groups vs. 3 × IT vehicle (day 69). Data plotted through point when ≥ 50% of animals in the group survived. Statistical significance was determined using one-way ANOVA with Dunnett’s post-test analysis. In all studies, red triangles designate days of treatment. Adapted with permission from [9]

Preclinical tumor studies in subcutaneous xenograft models of human renal cell carcinoma (786-O cell line (ATCC^®^ CRL-1932™) in Sprague–Dawley (Rag2/Il2rg null) SRG™ female rats), human transitional cell bladder carcinoma (UM-UC-3 cell line (ATCC^®^ CRL-1749™) in nude (Hsd:Athymic Nude-*Foxn1*^*nu*^) female mice), and human prostate carcinoma (PC-3 cell line (ATCC^®^ CRL-1435™) in nude (NCr *nu/nu* (Crl:NU(NCr)-*Foxn1*^*nu*^) female mice) demonstrated that up to 3 weekly cycles of IT LSAM-DTX resulted in tumor reduction or eradication, increased drug retention, and increased immune cell infiltrate into the tumor [9] (Table 2). In the renal cancer tumors implanted subcutaneously in immunodeficient female rats, 1, 2, and 3 cycles of weekly IT LSAM-DTX resulted in sustained tumor volume (TV) reduction relative to IT vehicle controls (Fig. 4a). In some animals where 3 cycles of IT LSAM-DTX were administered, complete eradication of tumor was observed. IV docetaxel failed to reduce renal tumor increase, with results similar to vehicle controls. IT LSAM-DTX treatments of bladder and prostate cancer implanted subcutaneously in immunodeficient female mice resulted in significant tumor reduction compared to IT vehicle control that was sustained until the study ended; tumors were reduced for at least 28 days (bladder, Fig. 4b) or 42 days (prostate, Fig. 4c) after the last LSAM-DTX administration. In the bladder cancer model, IV docetaxel treatment resulted in nondurable efficacy, with TV increasing approximately 10 days after the final cycle of IV chemotherapy.

**Fig. 5:**
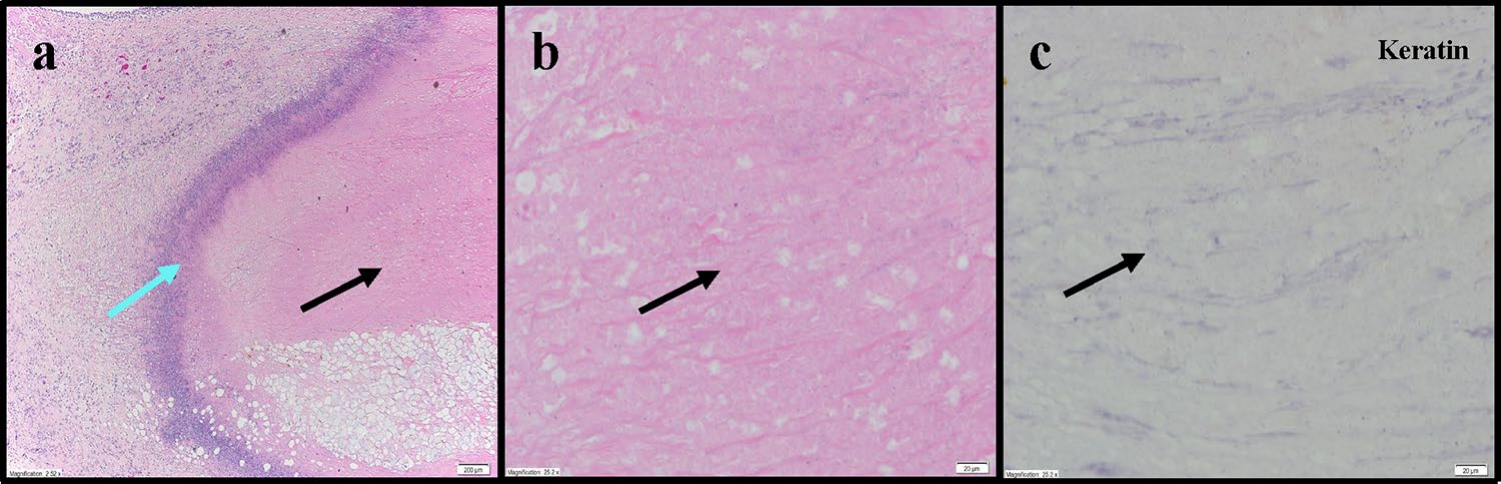
Representative photomicrographs of 786-O renal tumors administered three cycles of intratumoral (IT) large surface area microparticle docetaxel (LSAM-DTX). **a** Dense amorphous necrosis (black arrow) demarcated from surrounding fibrofatty tissue by a band of necrotic debris and admixed immune cells (blue arrow); 4 × magnification. **b** Necrotic area enlarged from panel **a**: no viable nucleated tumor cells (black arrow); 40 × magnification. **c** Necrotic area from panel **a**: absence of residual carcinoma evidenced by lack of cytokeratin (anti-AE1/AE3) staining (black arrow). Adapted with permission from [9]

In the renal cell carcinoma model, IT LSAM-DTX administration resulted in high levels of docetaxel within the tumor site tissues (up to 5 mg/g of tissue) up to 50 days following treatment, whereas docetaxel was undetectable in tumors from IV docetaxel–treated animals. Histopathologic review found that presence of residual carcinoma decreased with increasing cycles of IT LSAM-DTX administration. Extensive tumor cell necrosis was observed more frequently with increasing numbers of LSAM-DTX cycles. Pan-cytokeratin staining revealed foci of non-viable, anuclear, ghost tumor cell outlines consistent with labelling of degenerating keratin intermediate filaments in dead tumor cells (Fig. 5). In the bladder cancer model, histopathology of untreated tumors exhibited extensive diffuse proliferation of invasive carcinoma with markedly increased mitotic activity and scattered areas of coagulative necrosis. In contrast, tumor tissues from IT vehicle or IV docetaxel–treated animals demonstrated the same appearance as the untreated control tissue. In tumor implant sites collected from the animals treated with LSAM-DTX, 45% (5/11) had no residual viable carcinoma and 73% (8/11) showed extensive tumor cell necrosis and macrophage involvement.

### Abscopal effects in a syngeneic renal cell adenocarcinoma model

**Fig. 6:**
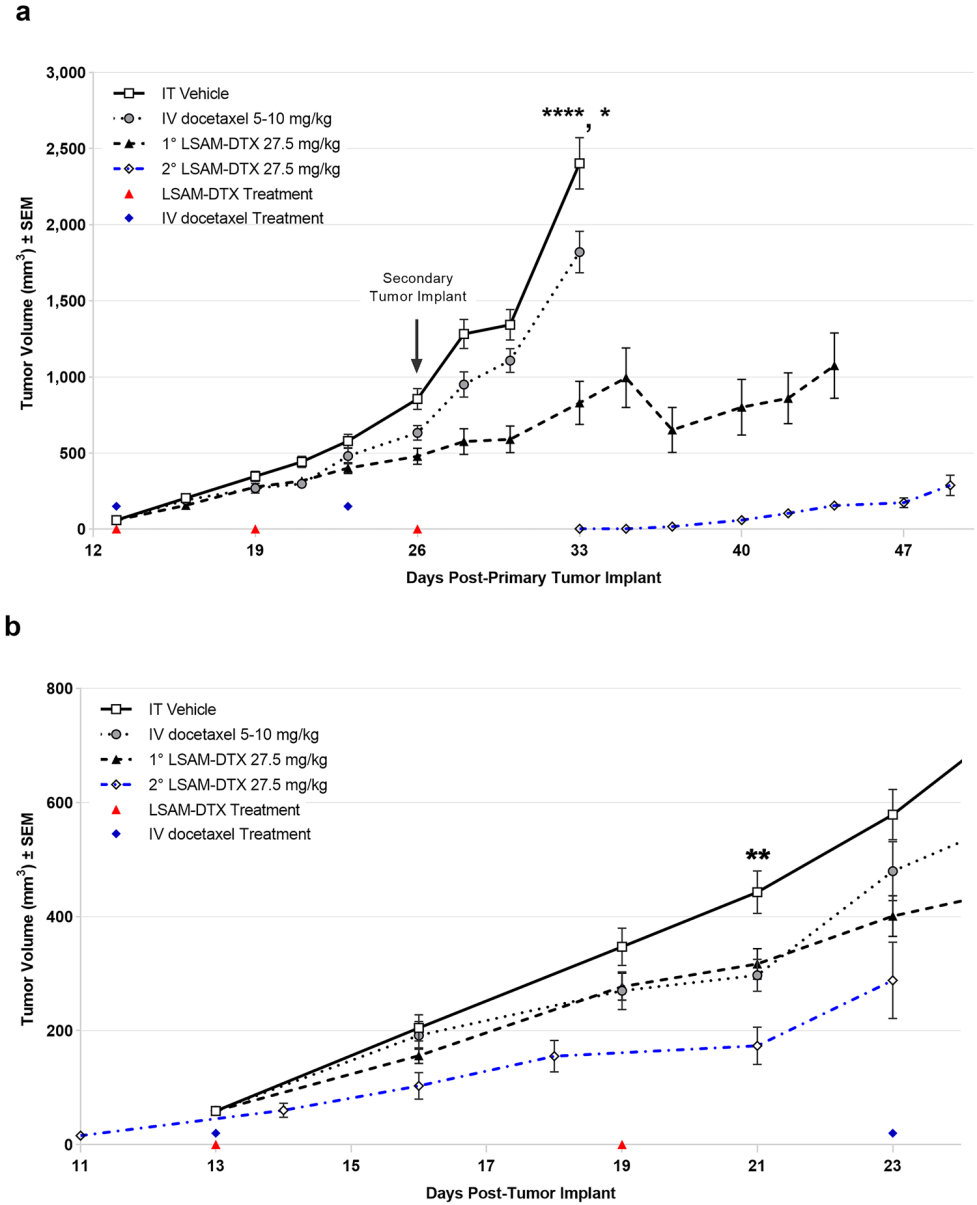
Intratumoral (IT) large surface area microparticle docetaxel (LSAM-DTX) is highly efficacious in reducing renal tumor growth. **a** Renca (ATCC^®^ CRL-2947™) xenografts in immunocompetent BALB/c (BALB/cAnNCrl) female mice; *n* = 15/group; treatment was initiated 13 days after implant when mean TVs = 58 mm.^3^. On day 26 following primary (1°) tumor implant on the right flank, animals received a second (2°) implant on the left flank. On day 33, 1° tumor volume (TV) in IT LSAM-DTX is significantly reduced vs. IT vehicle (****p < 0.0001) and IV docetaxel (***p < 0.001). Data plotted through point when ≥ 50% of animals in group survived. **b** Reduced TV growth in untreated 2° tumors in animals whose 1° tumors were treated with IT LSAM-DTX suggests an abscopal effect. 1° mean TV from days 13–23 after implant in animals treated with IT vehicle (*n* = 15), IV docetaxel (*n* = 15), or IT LSAM-DTX (*n* = 8: analysis restricted to the 53% of animals with 1° and 2° implants that survived through day 45) compared to untreated 2° mean TV from day 11 to day 23 after implant in the IT LSAM-DTX group (**p < 0.01; 2° LSAM-DTX vs IT Vehicle). Animals whose 1° tumors were treated with IT vehicle or IV docetaxel experienced rapid 1° tumor growth such that 100% reached trigger for humane sacrifice 7 days after second implant; prior to 2° tumor reaching detectable volume. Animals administered a single cycle of IV docetaxel at 10 mg/kg experienced body weight loss and adverse clinical signs that necessitated dose modification as follows: a delay in the second cycle from day 8 to day 11, second cycle dose reduction to 5.0 mg/kg, and cancellation of the third cycle. No dose modification was required for the LSAM-DTX-treated groups. Adapted with permission from [10]

The murine immunocompetent (syngeneic) model of mouse renal cell adenocarcinomas (Renca cell line (ATCC^®^ CRL-2947™) in BALB/c (BALB/cAnNCrl) female mice) was used to evaluate the effects of IT LSAM-DTX on tumor growth and local and peripheral immunomodulation (Table 2). Subcutaneously implanted tumors were treated with 3 weekly cycles of IT vehicle or IT LSAM-DTX or 2 weekly cycles of IV docetaxel. Flow cytometry was used to evaluate peripheral and tumor-associated immune populations [10]. Compared to untreated and vehicle or IV docetaxel-treated animals, tumor growth following IT LSAM-DTX was significantly reduced (Fig. 6a). Flow cytometry analysis in this immunocompetent model found that peripheral CD4 + , CD8 + , and Treg populations were significantly increased in bloods from animals administered LSAM-DTX compared to vehicle control.

A subset of animals was inoculated with a second Renca tumor at a distant site on the same day the final treatment of the primary tumor was administered. Animals remained on study until the combined primary and secondary TV reached 2000 mm^3^. Consistent with the results from the group bearing a single primary tumor, the LSAM-DTX-treated group had significantly reduced primary TV compared to vehicle and docetaxel. Combined primary and secondary tumor growth in animals treated with vehicle or IV docetaxel reached the maximum allowed volume and triggered euthanasia within 1 week of secondary tumor implant. Animals administered IT LSAM-DTX in the primary tumor had a reduced tumor growth rate in their untreated secondary tumors that was similar to IT LSAM-DTX treated tumors (Fig. 6b) and remained on study for 2 additional weeks.

These findings in the Renca model demonstrate that local LSAM-DTX treatment can more effectively reduce renal tumor growth compared to IV docetaxel therapy and enhance antitumor immunity. Flow cytometry evaluations showing increased CD4 + and CD8 + immune effector cells in the peripheral blood and the growth rate reduction in a secondary untreated tumor suggest that treatment of a primary tumor with IT LSAM-DTX may elicit abscopal effects which reduce the growth rate of distant untreated tumors.

### LSAM-DTX in combination with an immune checkpoint inhibitor in a metastatic breast cancer model

A syngeneic murine model of mouse metastatic stage IV breast cancer (4T1-Luc2-1A4 cell line (ATCC^®^ CRL-2539™) in BALB/c (BALB/cAnNHsd) female mice) was used to evaluate the anti-tumor and anti-metastatic activity of LSAM-DTX alone and in combination with immune checkpoint inhibitor therapy [11] (Table 2). 4T1 tumors are poorly immunogenic with minimal T cell infiltration that results in immunologically “cold” conditions in the TME where CD11b + myeloid cells and granulocytic myeloid–derived suppressor cells (G-MDSC) are the predominant infiltrating cell types [14]. 4T1 tumors can be implanted at the naturally occurring site of disease in the mammary pad (i.e., orthotopic implant) and, due to their highly aggressive and metastatic growth characteristics, will reproducibly metastasize to the thoracic cavity. The availability of luciferase-tagged 4T1 tumor cells allows for quantification of the extent of thoracic metastasis in-life using bioluminescence imaging (BLI).

The cytotoxic T lymphocyte antigen 4 (CTLA-4) is a critical receptor that regulates T cell activation and tolerance. CTLA-4 has been shown to inhibit T cell responses, and its blockade with the anti-CTLA-4 antibody leads to cell-mediated tumoricidal immunity [15, 16]. Mice implanted with 4T1 tumors can be systemically treated with the murine neutralizing antibody targeting CTLA-4 (anti-mCTLA-4) via intraperitoneal (IP) administration.

Tumor progression, onset, and extent of metastasis and immunomodulation were evaluated in 4T1 tumor–bearing mice treated with IT LSAM-DTX alone or in combination with IP anti-mCTLA-4 and compared to monotherapies or IT vehicle and untreated controls. Changes in body weight, TV, and BLI were evaluated over the following 24 days, at which point animals were sacrificed and immune cell populations in the primary tumor and peripheral blood were measured by flow cytometry.

**Fig. 7:**
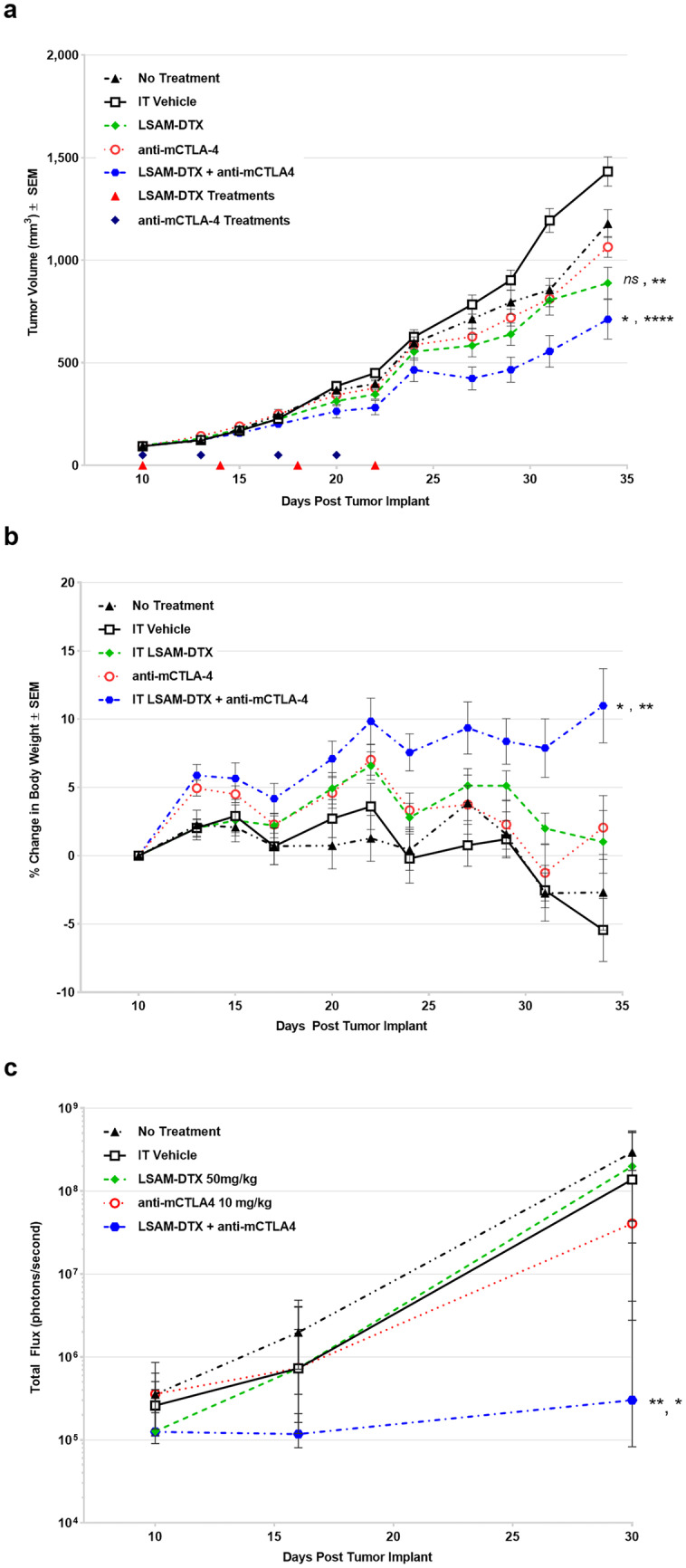
**a** Group mean tumor volume (TV) from treatment initiation on day 10 through end of study. *n* = 10 mice/group. All animals survived through day 34 with exception of 1 animal in untreated group which exited study on day 32. Large surface area microparticle docetaxel (LSAM-DTX; ~ 50 mg/kg) and vehicle were dosed intratumoral (IT) (red triangles). Anti-mCTLA-4 (10 mg/kg) was dosed IP (black diamonds). The combination group received both LSAM-DTX and anti-mCTLA-4 on the same schedules as the monotreatments. **b** Group mean percent change in body weight from treatment initiation through end of study. Error bars =  ± SEM. Significance reported for day 34 vs. no treatment, vehicle controls; *ns* = not significant, *p < 0.05, **p < 0.01, ****p < 0.0001. **c** Group median bioluminescence value (photons/second) ± IQR at days 10, 16, and 30. *n* = 10 mice/group. Significance reported vs. no treatment controls, LSAM-DTX monotherapy; *p < 0.05, **p < 0.01. Significance reported as *p < 0.05, **p < 0.01, ****p < 0.0001. Adapted with permission from [11]

This study found that IT LSAM-DTX significantly reduced breast cancer TV when given alone or in combination with anti-mCTLA-4. Compared to vehicle control, the greatest reduction in TV was achieved with the combination of IT LSAM-DTX + systemic anti-mCTLA-4 (Fig. 7a). Importantly, administration of the combination regimen was associated with significant animal weight gain (Fig. 7b) as well as lack of adverse clinical findings suggesting that LSAM-DTX may provide benefit in treatment regimens that include an immune checkpoint inhibitor without added systemic toxicity.

The LSAM-DTX + anti-mCTLA-4 combination significantly reduced 4T1 metastatic spread as measured by thoracic BLI (Fig. 7c). The combination of LSAM-DTX + anti-mCTLA-4 resulted in 40% of animals with no evidence of metastasis. Furthermore, a positive relationship was observed between reductions in tumor growth and metastatic spread.

**Fig. 8:**
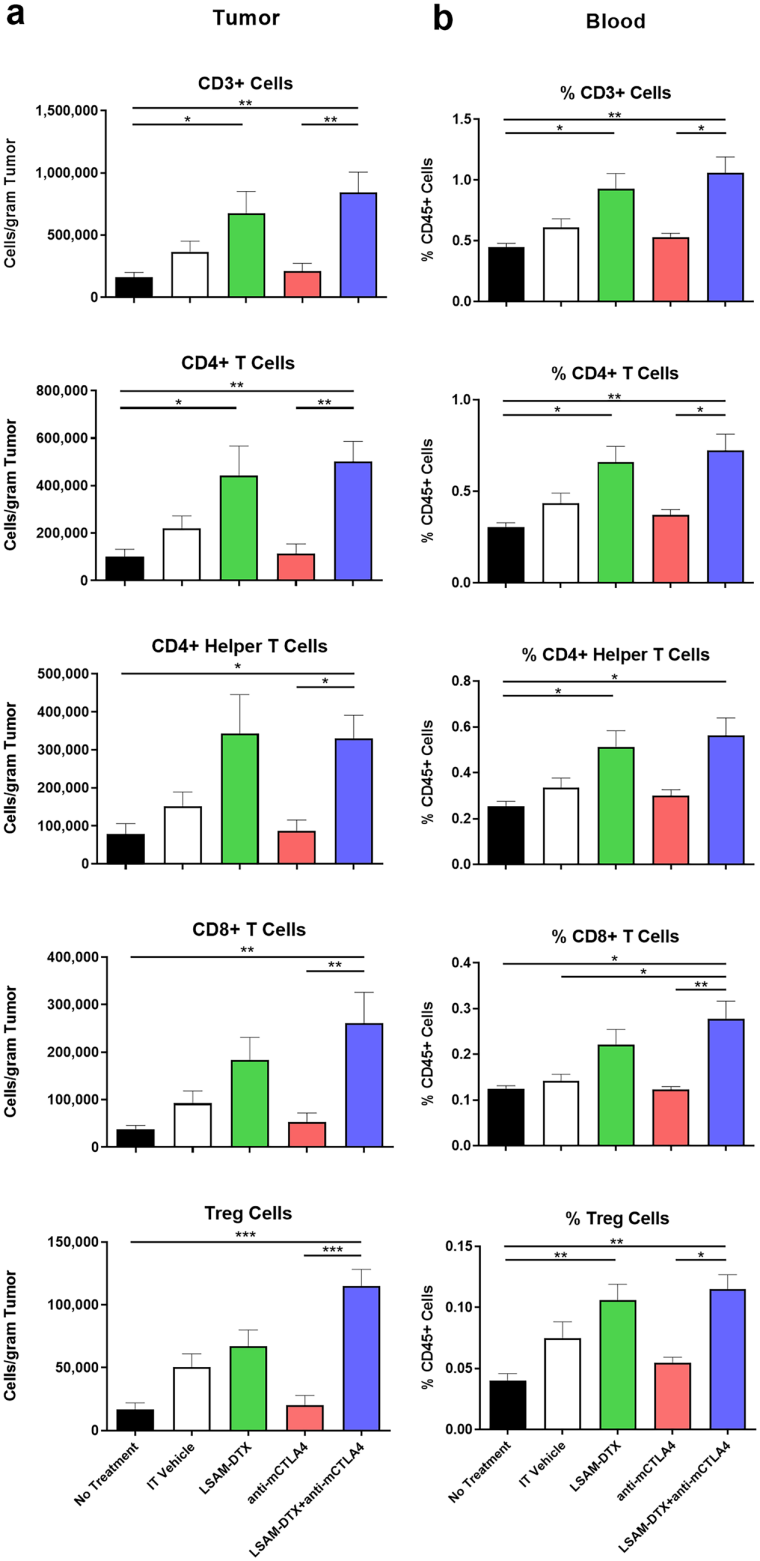
Intratumoral (IT) large surface area microparticle docetaxel (LSAM-DTX) treatment alone and in combination with systemic anti-CTLA4 therapy results in immunomodulation of the tumor microenvironment (TME) and peripheral blood in a metastatic breast cancer model. **a** Day 34 absolute counts of T cells in the TME (no treatment group *n* = 9 and mean tumor volume (TV) = 1179 mm^3^ and all other groups *n* = 10 and group mean TV range = 712 mm^3^ (LSAM-DTX + anti-mCTLA-4) to 1433 mm.^3^ (vehicle)); mean + SEM. **b** Day 34 percent of CD45 + cells stained as T cells in peripheral blood (*n* = 6 for no treatment group, *n* = 9 for anti-mCTLA-4 and *n* = 10 for all other groups); mean + SEM. Comparisons made using Kruskal–Wallis with Dunn’s multiple comparisons test. Significance reported as *p < 0.05; **p < 0.01; ***p < 0.001. Adapted with permission from [11]

Flow cytometry evaluations of tumors found that all categories of CD3 + T cells, including CD8 + cytotoxic T cells, were significantly increased in the combination group compared to untreated controls and anti-mCTLA-4 alone (Fig. 8a). The combination also increased the proportion of circulating T cells in peripheral blood, and IT LSAM-DTX alone resulted in increased populations of circulating CD3 + , CD4 + , and CD4 + helper T cells compared to untreated controls (Fig. 8b). Other local and circulating immunogenic cell types were affected by the combination of local LSAM-DTX and immune checkpoint inhibitor therapy, including increased density of natural killer (NK) cells, natural killer T (NKT) cells, and dendritic cells (DCs). The combination of IT LSAM-DTX and anti-mCTLA-4 significantly reduced absolute counts of immunosuppressive circulating myeloid-derived suppressor cells (MDSCs).

IT LSAM-DTX administered with systemic anti-CTLA-4 reduced primary breast cancer TV and reduced or eliminated metastatic spread without added systemic toxicity. Flow cytometry evaluations show that the combination treatment converted the TME from immunogenically “cold” to “hot” as well as increased circulating antitumor immune cells and decreased immunosuppressive MDSCs.

## Clinical trials

### Bladder cancer

An open-label 3 + 3 dose-escalation clinical trial evaluated the safety and tolerability of LSAM-DTX in subjects with high-risk NMIBC or MIBC (NCT03636256) (Table 1). LSAM-DTX was given as an intramural direct injection circumferentially around the resection site immediately following transurethral resection of bladder tumor (TURBT) and as intravesical therapy. Subjects were evaluated for safety and tumor recurrence or disease progression up to 12 months from first administration of LSAM-DTX. Plasma samples were collected at timepoints throughout the study to characterize the pharmacokinetics (PKs) of docetaxel. Tissue was collected for multiplex immunofluorescent (mIF) and quantitative immunophenotyping.

#### High-risk non-muscle invasive bladder cancer

Subjects (*n* = 19) with high-risk NMIBC received direct injection of LSAM-DTX following standard of care (SOC) TURBT. Subsequently, subjects received intravesical instillation of LSAM-DTX within 2 h of the direct injection. After a ≥ 4-week recovery period, subjects proceeded to an induction period followed by a maintenance period [12]. Direct injection of LSAM-DTX followed standard dose escalation (0.75, 1.5, 2.5, or 3.75 mg/mL) with 6 additional subjects receiving the high dose (3.75-mg/mL) in an expansion cohort (Table 3). Intravesical instillation of LSAM-DTX was escalated per subject from 50 to 75 mg, following 3 weekly induction cycles.

**Table 3:** Summary of large surface area microparticle docetaxel (LSAM-DTX) suspension concentrations and total dose administered for the high-risk non-muscle invasive bladder cancer study. Adapted with permission from [12]

		**Direct-injection LSAM-DTX**^**a**^		**Intravesical LSAM-DTX**^**b**^		
**Cohort**	***n***	**Conc. of injection suspension (mg/mL)**	**Total LSAM-DTX delivered (mg)** **(mean, range)**	**Conc. of induction**^**c**^**, maintenance**^**d**^ **suspension (mg/mL)**	**No. of instillations (mean; range)**	**Total LSAM-DTX delivered (mg)** **(mean; range)**
1	4	0.75	3.00	2.00, 3.00	10	645 (630–650)
2	3	1.50	4.50 (3.75–6.00)	2.00, 3.00	9 (7–10)	625 (510–714)
3	3	2.50	7.92 (6.25–10.00)	2.00, 3.00	9 (7–10)	575 (425–650)
4	3	3.75	15.00	2.00, 3.00	9 (8–10)	600 (500–650)
Expansion	6	3.75	14.06 (9.38–15.00)	3.00^*e*^, 3.00	10	746 (725–750)

^a^Direct injection of LSAM-DTX into the index tumor resection site on the bladder wall occurred once immediately following transurethral resection of bladder tumor (TURBT)

^b^Intravesical administrations of LSAM-DTX occurred up to 10 times: once within 2 h of the TURBT/direct injection and up to 9 times during the induction and maintenance phases

^c^Induction phase was initiated ≥ 4 weeks post-TURBT and included once weekly instillations for up to 6 weeks

^d^Maintenance phase was initiated ≥ 7 weeks after the start of the induction phase and included once weekly intravesical administration for up to 3 weeks

^e^One subject received a single maintenance intravesical therapy cycle at 2.00 mg/mL LSAM-DTX

Thirty-one local treatment-emergent adverse events (TEAEs) occurred on the study with one instance of hematuria considered probably related. Additionally, there were 30 possibly related events that occurred that most commonly included hematuria, dysuria, and urinary tract infection. No drug-related local or systemic serious adverse events (SAEs) were reported. PK samples collected throughout the study found negligible systemic docetaxel concentrations in all samples.

**Fig. 9:**
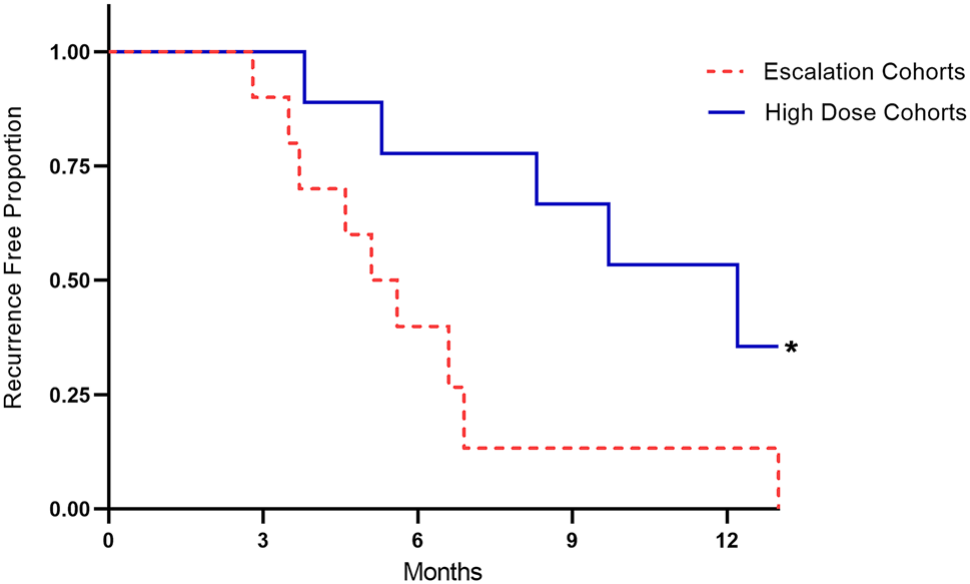
Recurrence free survival (RFS) as a proportion of high-risk non-muscle invasive bladder cancer (NMIBC) subjects in the escalation cohorts 1–3 (*n* = 10; dotted line) vs. the high dose and expansion cohorts (solid line). Median RFS in the low dose escalation cohorts = 5.4 months vs. 12.2 months in the high-dose and expansion cohorts. Significance (*p < 0.05) determined using Log-rank Mantel-Cox test; hazard ratio (Mantel–Haenszel) = 0.29 with 95% CI = 0.09–0.88. Adapted with permission from [12]

In the low doses of the escalation cohort, the median follow-up time was 8.6-months. Recurrence occurred in 9 of 10 subjects (90%) at a median of 5.4 months and the estimated recurrence free survival (RFS) at 3 and 6 months was 90% and 40%, respectively. In the high-dose escalation cohort and the expansion cohort, the median follow-up time was 12.4 months. Recurrence occurred in 5 of 9 subjects (56%) at a median of 12.2 months and the estimated RFS at 3, 6, and 12 months was 100%, 78%, and 50%, respectively (Fig. 9). In the high-dose escalation cohort and the expansion cohort, LSAM-DTX was associated with significantly increased RFS compared to lower doses (hazard ratio 0.29, *p* < 0.05).

**Fig. 10:**
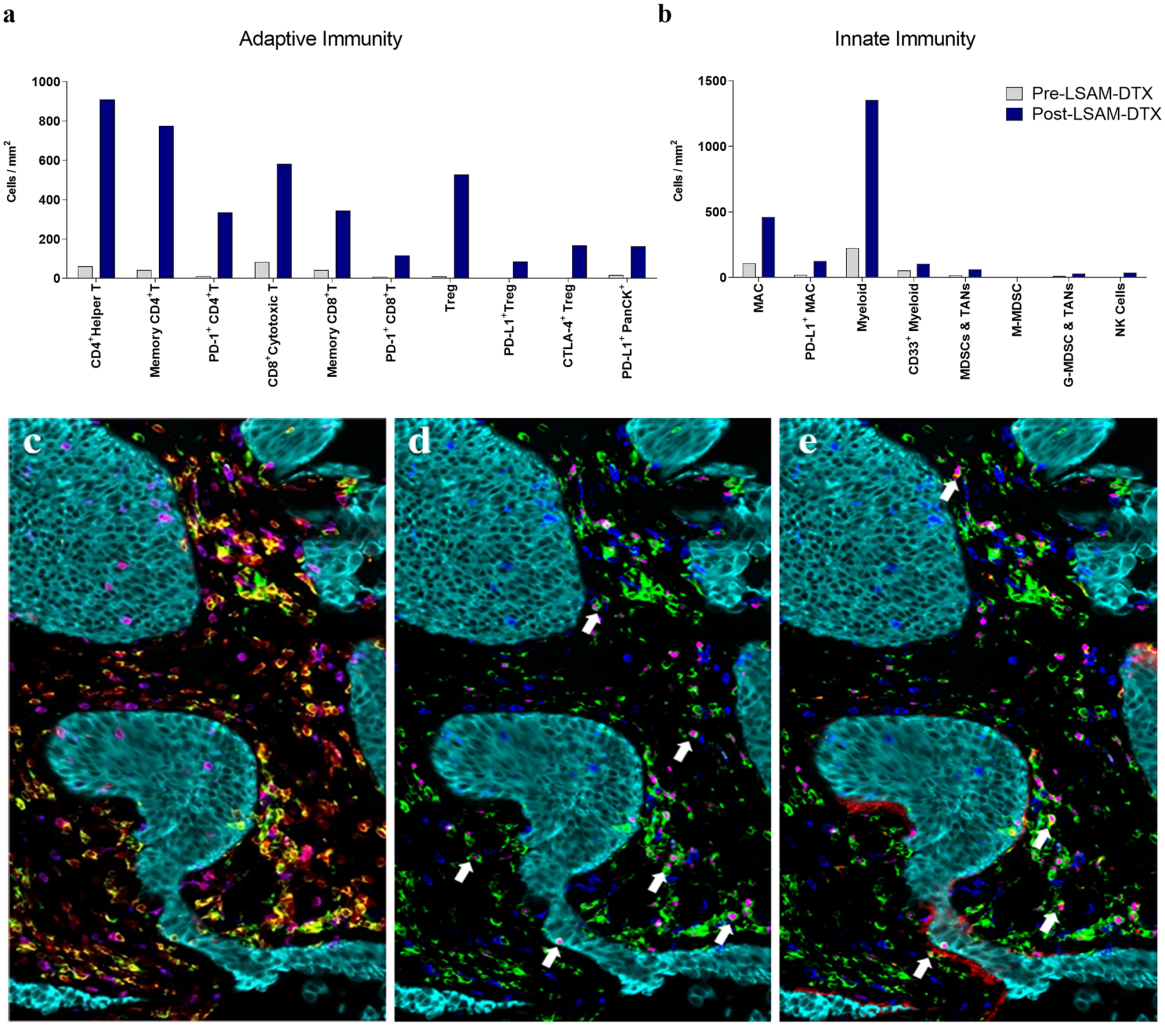
mIF data from bladder biopsies collected pre- and post-LSAM-DTX therapy in a BCG-naïve high-risk NMIBC patient who had no prior TURBT procedures. Patient had complete response (CR) at 12 months and showed increases in post-LSAM-DTX density of **a** adaptive and **b** innate immune cells including increases in the immune checkpoint inhibitor targets PD-1 (increased on CD4^+^ helper T, CD8^+^ cytotoxic T), PD-L1 (increased on Treg, macrophages, and PanCK.^+^ cells), and CTLA-4 (increased on Treg) compared to pre-LSAM-DTX (collected at TURBT). (c–e) Representative color overlays of ROI from post-LSAM-DTX tumor biopsy showing high density of effector T cells in the TME. Cells are stained as follows: **c** yellow = helper t cells (CD3 + CD4 +), magenta = cytotoxic T cells (CD3 + CD8 +), light blue = PanCK + tumor and bladder cells; **d** white arrows show examples of T reg cells; green = CD4 + , magenta = FoxP3 + , dark blue = CD8 + , light blue = PanCK + tumor and bladder cells; **e** white arrows show examples of T reg cells expressing CTLA-4; green = CD4 + , lavender = FoxP3 + , red = CTLA-4 + , dark blue = CD8 + , light blue = PanCK = cells. Adapted with permission from [12]

**Fig. 11:**
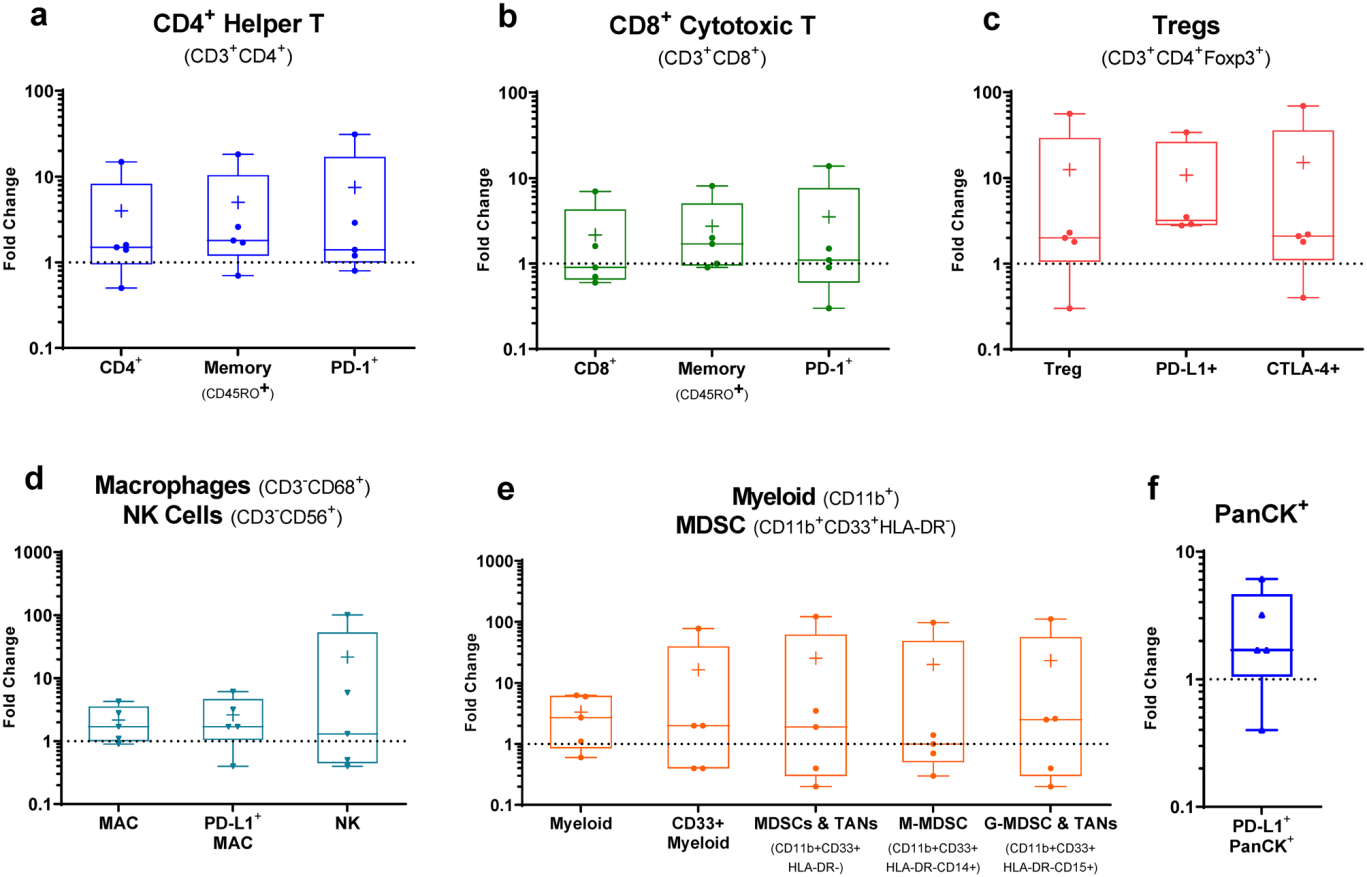
Changes in immune cell density in NMIBC subjects fold change in immune cell densities (cells/mm^2^) from biopsies collected before and after LSAM-DTX therapy as determined by mIF. Boxplots show min. to max. fold changes (extremes), per subject data (dots), mean ( +), and median (line) for *n* = 5 subjects analyzed. Cell types are identified using marker co-expression over an average of 24 regions of interest (ROI; range = 7 to 37) within the TME per slide. ROI selected by a pathologist blinded to treatment-status on a hematoxylin and eosin-stained slide. Cell type density is the total number of cells counted divided by the total area (sum of all ROI areas) per slide. In cases where the pre-LSAM-DTX cell density was 0 cells/mm^2^, no data is reported and *n* < 5. **a** CD4^+^ Helper T cells. **b** CD8^+^ cytotoxic T cells. **c** Treg cells. **d** Macrophage (MAC) and NK cells. **e** Myeloid and MDSC cells; MDSC and tumor-associated neutrophils (TANs). (**f**) PanCK^+^PD-L1.^+^. Adapted with permission from [12]

mIF data show favorable adaptive (Fig. 10a) and innate (Fig. 10b) immune cell infiltration following LSAM-DTX treatment in Bacillus Calmette-Guérin (BCG)-naïve subjects from whom pre- and post-LSAM-DTX bladder biopsy tissue was available. Interestingly, substantial increases in immune checkpoint therapy targets such as PD-L1 + tumor cells, PD-L1 + Tregs, and CTLA-4 + Tregs (Fig. 10c–e) were found. In addition, across 5 subjects with biopsies collected pre- and post-LSAM-DTX therapy, mIF analysis of the TME found increases in density of immune effector T cells, NK cells, and immune checkpoint inhibitor targets (Fig. 11).

#### Muscle invasive bladder cancer

In the same open-label 3 + 3 dose-escalation clinical trial that evaluated subjects with high-risk NMIBC (NCT03636256), a total of 17 subjects with MIBC were enrolled. Median subject age for MIBC subjects was 79 years, 82% were male and 88% identified as White. Subjects received direct injection of LSAM-DTX following SOC TURBT, followed by an initial intravesical instillation of LSAM-DTX within 2 h of the direct injection. Subjects then went on to receive SOC. In subjects with MIBC, direct injection and intravesical administration of LSAM-DTX followed the same dose escalation previously described for high-risk NMIBC, with 5 additional subjects enrolled at the highest dose.

Subjects were evaluated for safety and preliminary efficacy for 45 days and then proceeded to institutional SOC and did not receive additional LSAM-DTX. Both direct injection and intravesical administration of LSAM-DTX were well tolerated without drug-related systemic or local SAEs. Seventy-seven TEAEs were reported. Of these, 11 possibly related local TEAEs occurred in 5 subjects. No systemic TEAEs were considered related to LSAM-DTX. PK analysis demonstrated negligible systemic concentrations of docetaxel. Docetaxel was detected slightly above the lower limit of quantitation only at 1 and 4 h post-injection of LSAM-DTX.

## Summary and conclusions

The maximum tolerated doses of IV chemotherapies are often limited by toxicities such as cytopenia due to bone-marrow suppression [17]. In contrast, local administration of chemotherapy allows for a substantial reduction in systemic exposure and drug-related systemic adverse events [2, 12, 18]. Clinical results of systemic combinations of chemotherapy and immunotherapy have often been disappointing [19]. The lack of synergism with the systemic therapies may be explained by depletion of lymphocytes due to IV chemotherapy-related bone marrow suppression [20–22].

When IT LSAM-DTX was evaluated in preclinical uro-oncologic models, docetaxel was detectable in tumor tissue up to 50 days post-IT LSAM-DTX administration [9]. The extended residence time of IT LSAM-DTX in the TME may induce necroptotic tumor cell death and enhance permeability of the tumor’s stromal matrix facilitating distribution and availability of neoantigens and docetaxel within the tumor. The increased availability of neoantigens could further facilitate tumor cell immunogenic cell death [19, 23].

In a bilateral syngeneic renal carcinoma tumor model, abscopal-like effects were observed in the reduction of growth in an untreated secondary tumor in which the primary tumor was previously treated with IT LSAM-DTX [10]. IT LSAM-DTX injections into syngeneic murine breast tumors reduced TV and, in combination with anti-CTLA-4, further reduced primary tumor growth and extent of thoracic metastasis. These data are consistent with the hypothesis that the necroptotic response of tumor cells may allow an influx of immune effector cells into the TME [24].

Cytotoxic T lymphocytes, NKT cells, and NK cells represent the major cytotoxic cell subsets involved in tumor cell death. Elevation in peripheral blood concentrations of effector T and NK cells and reductions in suppressor cells were shown in pharmacology studies reviewed here [10, 11]. Increased immune cell concentrations in the TME and their juxtaposition to viable tumor cells, as seen in the mIF analysis in tumor samples from high-risk NMIBC subjects treated with LSAM-DTX, may be due to their enhanced access following LSAM-DTX-related alterations in the stroma.

NK cells typically arrive early in the TME and, together with DCs, can facilitate an effective T cell tumoricidal response [25–27]. Following IV treatments, NK cell levels in the TME are typically limited by an inability to accumulate in the tumor [28]. In the metastatic breast cancer model, the combination of LSAM-DTX + anti-CTLA-4 resulted in significant decrease in TV and thoracic metastasis and increased T cells, NK cells, and NKT cells in the tumor site and peripheral blood. These effects may be related to local release of tumoricidal cytokines by large numbers of NK and T cells [29–31]. Tumoricidal contributions of NK cells, cytotoxic T cells, and NKT cells in the immune response to many forms of breast cancer [32–34] could be enhanced by IT administration of LSAM-DTX into the primary tumor followed by systemic administration of immunotherapy.

Direct injection and intravesical administration of LSAM-DTX were well tolerated in preclinical and clinical studies [9–12]. In a model of metastatic breast cancer, the combination of IT LSAM-DTX and a systemic immune checkpoint inhibitor resulted in increased weight gain and lack of negative systemic side effects often associated with chemotherapy or immunotherapy. No events of severe systemic toxicity were attributed to LSAM-DTX in our study of high-risk NMIBC and MIBC, which is supported by the negligible levels of docetaxel in the peripheral blood [12]. No local SAEs and only 1 local TEAE probably related to LSAM-DTX (hematuria, which is not unusual following catheterization) were reported. These findings suggest that LSAM-DTX may be safe for use in combination with other local or systemic therapies to improve response.

Recurrence of high-risk NMIBC puts the patient at risk [35, 36]. The most common site of recurrence is the initial site of disease presentation. Direct injection of LSAM-DTX into and around the resection site following TURBT is designed to reduce residual tumor cell survival in the intramural portion of the bladder around the resection site. Results of the LSAM-DTX clinical trial confirm and extend reports of intravesical docetaxel treatment of high-risk NMIBC alone and with the addition of gemcitabine [37, 38]. The clinical response data provide evidence that local injection may further reduce local recurrence, especially in stage Ta and T1 lesions in which disease penetrates through the superficial urothelium.

LSAM-DTX given both by direct injection and by intravesical therapy may provide the urologist with a useful addition to bladder cancer treatment with the aim of bladder retention, without adding significant systemic toxicity. Primary or recurrent papillary disease, histological variants, and discrete CIS lesions (Ta and T1), high-risk NMIBC, and MIBC stage T2 as well as trimodal therapy for MIBC [39] may all benefit from direct injection and intravesical administration of LSAM-DTX.

Previous investigations in bladder cancer have found that stromal immunophenotype that includes increased cytotoxic T cells and NK cells, similar to those described in subjects treated with LSAM-DTX, is predictive of improved overall survival and disease-free survival [40]. Based on synergy shown with IT LSAM-DTX followed by systemic anti-CTLA-4 in the 4T1 study summarized here [11], injection of LSAM-DTX in TURBT resection sites followed by intravesical anti-CTLA-4 may also extend RFS in patients with NMIBC.

Additional opportunities may also exist for use of LSAM-DTX as IT neoadjuvant therapy in patients undergoing cystectomy with or without systemic immunotherapy including use with radiation therapy in patients with more advanced bladder cancer. Larger, multicenter, follow-on trials are in the planning stage to confirm the clinical benefit of LSAM-DTX in various genitourinary malignancies. Other areas of clinical interest include breast cancer and melanoma, both of which have shown to be sensitive to IT LSAM-DTX.

Engineering of large surface area microparticle taxanes together with advances in IT delivery systems has allowed for localized treatment of solid tumors without addition of dose-limiting toxicity. The preclinical and clinical results from studies of IT LSAM-DTX reviewed here, and our prior publications of investigations of IT LSAM-PTX, demonstrate feasibility of this approach to improve cancer treatment. Ongoing development of other chemotherapies and targeted therapies for IT delivery alone and in combination with currently available treatments may represent an important contribution to patient care [19, 41–44].

## Data Availability

The datasets generated during and/or analyzed during the current study are available from the corresponding author on reasonable request.
